# Microbiological Aspects of Acute and Chronic Pediatric Rhinosinusitis

**DOI:** 10.3390/jcm8020149

**Published:** 2019-01-28

**Authors:** Lorenzo Drago, Lorenzo Pignataro, Sara Torretta

**Affiliations:** 1 Clinical Microbiology, University of Milan, 20100 Milan, Italy; lorenzo.drago@unimi.it; 2 Fondazione IRCCS Ca’ Granda Ospedale Maggiore Policlinico, 20100 Milan, Italy; lorenzo.pignataro@unimi.it; 3 Department of Clinical Sciences and Community Health, University of Milan, 20100 Milan, Italy

**Keywords:** Children, rhinosinusitis, paranasal sinuses, infection, microbiology, biofilm

## Abstract

The microbiology of acute and chronic rhinosinusitis has been extensively studied, but there are still some differences of view concerning the etiology of the former, and many disagreements regarding the microbiology of the latter. Establishing the concomitant distribution of the causative micro-organisms in cases that involve multiple sinuses is scientifically and practically important. The main problems are the variety of aerobes and anaerobes that may be involved, and the fact that different tracts of the sinuses of the same patient may be simultaneously affected. Rhinosinusitis may also involve the formation of biofilm, which may play a significant role in its pathogenesis and persistence. Biofilms have a number of advantages in terms of bacterial survival, and their perpetuation can create a certain degree of instability in host-bacteria interactions. Sinonasal microflora may further complicate pathogenesis and the identification of the pathogen(s) involved. Furthermore, the concentration, uniformity, and type/number of strains of nasal microbiota may vary from one site to another. The relative and total micro-organism counts can also be affected by various factors, and microbiota can modulate the course of both acute and chronic rhinosinusitis.

## 1. Introduction

Acute and chronic rhinosinusitis (ARS and CRS) are among the most frequent problems facing healthcare professionals that can evolve to complicated disease requiring urgent treatment. Despite extensive research, no precise bacterial and/or viral etiology has yet been established. The conclusion of studies carried out in the 1990s was that “despite the substantial prevalence and clinical importance of sinusitis of childhood, there has been relatively limited study of the microbiology of sinusitis in pediatric patients” [1,2]. However, since then, a number of studies have clarified various microbiological aspects of rhinosinusitis, including its epidemiology, the role of bacterial biofilm producers, and recently the microbioma of healthy sinuses. 

Nevertheless, knowledge is still limited, partly because of the relative inaccessibility of the paranasal sinuses. Unlike middle ear cavities, paranasal sinuses cannot be inspected directly, and their aspiration is more difficult and less frequently undertaken than in other cases such as tympanocentesis. Furthermore, in order to be able to determine the bacteriological cause of an infected sinus, it is necessary to obtain a secretion sample that is not contaminated by the normal respiratory or oral flora colonizing mucosal surfaces.

Bacterial biofilm can be considered an “influencer of infections” and a new super-organism, especially in the case of sinusitis. More than 99% of bacteria live in the thinly layered colonies known as biofilms, which can cause serious, chronic, and intractable infections. The bacteria inside a biofilm can communicate with each other by means of molecular mechanisms that trigger some cells to evade treatment by lying dormant, and others to die for the benefit of the colony [3]. An ability to disrupt these signals could lead to promising new treatments. 

Many scientists consider bacteria with or without biofilm to be “social organisms” that form the so-called microbiota: i.e., well-structured or disorganized microbial communities that respectively ensure the health or otherwise of bodily districts such as the respiratory tract, intestines or vagina. The recent development of metagenomics based on next-generation sequencing (NGS) has improved our knowledge of the intricate microbial ecology of the paranasal sinuses. As the composition, distribution and abundance of microbiota have an impact on mucosal health and can influence the growth and function of pathogens, a greater understanding of host-microbiome constituents and relationships may encourage the development of new treatments for acute or chronic rhinosinusitis that can restore microbiome homeostasis and the cultivation of optimal microbial communities [4]. Understanding the role, relationships and epidemiological evolution of micro-organisms is vitally important. Significant differences in the microbiology of CRS and ARS have emerged since the 1990s, and now allow the knowledge-based selection of appropriate empirical treatments.

The aim of this article is to review the studies that have delineated the microbiology of acute and chronic pediatric sinusitis by providing further information about their general epidemiology and the role of biofilm and microbiota modulation in negative and positive disease outcomes. 

## 2. Historical Epidemiology of Pediatric CRS

In 1992, Ellen R. Wald found large amounts of bacteria in 70% of children with acute sinusitis, persistent or severe symptoms, and abnormal sinus radiographs [2]. The most frequent micro-organisms were *Streptococcus pneumoniae*, closely followed by *Moraxella catarrhalis* and *Haemophilus influenzae* which, as they can also produce beta-lactamase, are amoxicillin resistant. The *H. influenzae* strains were almost always untypeable and, rarely, type b. Only one anaerobic bacterial species (*Peptostreptococcus*) was isolated, and viruses were infrequent (Box 1).

Box 1 **Epidemiology of acute rhinosinusitis (ARS).**

1990s: Early-stage viruses, secondary infection due to aerobic bacteria (*S. pneumoniae*, *H. influenzae* and *M. catarrhalis*).

2018: *S. pneumoniae*, *M. catarrhalis*, *H.influenzae*, beta-hemolytic streptococci, *Staphylococus aureus*. Polymicrobial flora is present in about one-third of cases, and anaerobes (still under-diagnosed) are often associated with dental infections.

Agreement concerning the epidemiological structure of acute sinusitis was reached in the 1990s, but the results of the studies of CRS carried out in the same period were conflicting because of differences in the methods of collecting and, especially, quantitatively culturing sinus secretions. Brook et al. [1] demonstrated that aerobes (particularly staphylococci and streptococci) were isolated in approximately 38% of cases, whereas *Haemophilus* species were rare. The most frequent bacteria were anaerobic Gram-positive cocci and a large group of *Bacteroides* species, especially *B. melaninogenicus*, and fusobacteria, whereas other studies [5,6] found that the predominant bacterial isolates were *H. influenzae*, *S. pneumoniae*, *M. catarrhalis*, *group A Streptococcus,* and *S. aureus*. The study of Brook et al. was also probably the first to show that the presence of anaerobes in the sinus is related to disease duration and severity (Box 2).

Box 2 **Epidemiology of chronic rhinosinusitis (CRS).**

1990s: Staphylococci and streptococci (a small percentage of *Haemophilus*), but especially anaerobes (*Peptostreptococcus*, *Bacteroides* and fusobacteria)

2018: *Staphylococcus aureus* and anaerobic organisms (*Prevotella*, *Porphyromonas*, *Fusobacterium*, and *Peptostreptococcus spp*.). One-third of the aerobic and anaerobic bacteria produce beta-lactamase, whereas 60% of *S. aureus* are methicillin resistant. *Pseudomonas aeruginosa* and other aerobic and facultative gram-negative rods are frequently recovered in patients with co-morbidities.

Rhinosinusitis is still often under-diagnosed in pediatric practice, but a diagnosis of both ARS and CRS can be made on the basis of their history and symptoms. We now know that the early phase of ARS may be viral, usually lasts for up to 10 days, and ends with the complete recovery of most patients. However, some patients develop an acute bacterial infection generally caused by facultative aerobic bacteria (e.g. *Streptococcus pneumoniae*, *Haemophilus influenzae* or *Moraxella catarrhalis*) and, if the acute infection continues, anaerobic bacteria originating from oral flora can become the predominant pathogens. These phases have been demonstrated in patients with maxillary sinusitis by making serial cultures throughout the period of infection and in a recent cohort described in the Pediatric Infectious Disease Journal [7,8] (Box 1).

The presence and the percentage of these micro-organisms can be influenced by previous anti-microbial treatments, vaccinations, the presence of normal flora capable of interfering with the growth of pathogens, geography, and site (the maxillary, ethmoid and frontal sinuses show different etiologies) [9]. In patients with ARS who had micro-organisms simultaneously isolated from two sinuses, it was found that 56% were found in only one and 44% in both whereas, in those with chronic infection, 34% were recovered from one sinus and 66% from both. Anaerobic bacteria were isolated from the two sinuses in 80% of cases, and aerobic and facultative bacteria in 50%. These data underline the importance of obtaining cultures from all of the infected sinuses of patients with infections in more than one [10]. 

CRS is now considered a multifactorial disease process in which it is believed that bacteria play a role in propagating inflammation. Recognition of the etiological agents in such cases is very important when selecting anti-microbial therapy (Box 2). There is a reasonable consensus concerning the selection of a first-line antibiotic and the duration of treatment in children with CRS, although there are no recently published prospective studies of antibiotics. The methods of diagnosing CRS need to be appropriately selected, and should include a metagenomic approach to evaluate the nasal microbiome, especially in refractory cases. A patient’s age can also play an important epidemiological role as the disease in older adolescents can be similar to adult CRS [11]. 

A multi-center study by Ivanchenko et al. [12] had the aim of identifying the micro-organisms inhabiting the maxillary sinus and middle nasal meatus in patients with CRS. Aerobes were more frequent than anaerobes in both the nasal cavity (78.7% vs. 21.3%) and the maxillary sinus (55.2% vs. 44.8%), in which *Streptococcus spp* (28.8%) and *Prevotella* (17.8%) were the most frequent, whereas *S. pneumonia*, *H. influenzae*, and *S. aureus* were relatively rare (respectively 6.7%, 5.4%, and 8.9%). Although the study involved adults, its findings give an idea of the wide range of micro-organisms which can be isolated in CRS patients. There are unfortunately very few similar studies of children, partially because children with CRS have usually been previously treated with antibiotics, which often makes it difficult to distinguish bacterial flora from pathogenic agents. The studies mentioned above generally used different collection techniques, which may explain why Mantovani et al. found no bacterial growth in 53.2% of their patients with CRS, with 45.2% of aerobic bacteria in the positive cultures and no anaerobic bacteria: the most frequent aerobe was *Pseudomonas aeruginosa* [13].

A subsequent study by Brook et al. published in 2006 [14] found that the micro-organisms isolated from patients with acute exacerbations of CRS were mainly anaerobic. This is similar to those usually found in patients with CRS, whereas aerobic micro-organisms such as *H. influenzae*, *M. catarrhalis*, and *S. pneumoniae* are generally found in the case of acute infections and may also appear during some acute exacerbations of CRS.

As these studies reflect different epidemiological scenarios, it is difficult to use their data when approaching the choice of antibiotic treatment. However, on the basis of the etiological agents found in CRS, Brook [15] recently suggested an initial, empirical selection of anti-microbial agents effective against the most likely aerobic (*S. pneumoniae*, *H. influenzae*, and *M. catarrhalis*) and anaerobic bacterial pathogens, (*Fusobacterium nucleatum*, pigmented *Prevotella*, *Porphyromonas*, and *Peptostreptococcus spp*), especially in the case of community-acquired infections, while also covering methicillin-resistant *S. aureus* (MRSA).

## 3. Biofilms in Pediatric CRS: Lessons from Adult Sinusitis

Bacteria can naturally be found as free-floating planktonic or sessile cells known as biofilms, an adaptive phenotypic switch of prokaryotic life characterizing all living bacteria, and the main mode of bacterial survival and proliferation. [16]. 

Although much evidence suggests that there is a link between biofilms and CRS in humans, little is known about their real contribution to the pathophysiology of the disease as there are still no definitive studies demonstrating the factors that determine their persistence and growth on the sinonasal mucosa of the host, especially in children.

There is no doubt that intra-mucosal bacterial micro-colonies exist in CRS but they do not necessarily induce a local immune response. Although the pathogenesis of CRS is still uncertain, recent studies have identified polymicrobial biofilms on the surface of sinus mucosa and *Staphylococcus aureus* in the sinus mucosa of patients with CRS [17] (Box 3).

Box 3 **Biofilms in rhinosinusitis.**

A number of aerobes and anaerobes are capable of producing biofilms, which differ depending on the micro-organism and stimulate various inflammatory molecules during acute or chronic rhinosinusitis. The most negative features of biofilms is their high degree of resistance to antibiotics and host immune mechanisms as they are less susceptible to opsonisation and phagocytosis.

Pagella et al. [18] studied rhinosinusitis and otitis media in children by investigating hypertrophic adenoidal tissue, and concluded that the presence of bacterial biofilms on adenoids suggests that they are responsible for both diseases. Furthermore, Coticchia et al. [19] clearly demonstrated the presence of biofilm in the nasopharynx of children with CRS in comparison with those affected by obstructive sleep apnea, and Kania et al. [20] showed that many micro-organisms and species in the respiratory tract (*Corynebacterium argentoratense*, *Streptococcus spp*, *Micrococcus luteus*, *Staphylococcus aureus*) can easily produce biofilms.

However, despite this clear evidence of the role of biofilm in rhinosinusitis, other authors (Mladinaa and Skitarelić) sustain the hypothesis that biofilm in the nose and paranasal sinuses is nothing else but the normal respiratory mucosal blanket containing a variable number of bacteria and part of the mucociliary system itself [21].

It is likely that biofilms occur in many cases of CRS, but the significance of their detection in some series should be carefully considered as well as the methodology used for processing clinical samples [22]. Torretta et al. clearly showed that nasopharyngeal swabs seem to be less accurate in detecting biofilms in children than a biopsy [23].

Given the difficulty of studying biofilms in a viable tissue culture or even in animals, only few studies have evaluated their interactions with the host [24,25]. 

Other studies have highlighted the microbiological features of pathogens producing biofilm. Tapiainen et al. found that serotypes 33 and 14 of *S. pneumoniae* were the most efficient in forming biofilms in pediatric patients, whereas serotypes 3 and 38 were poor producers [26]. 

One particular mechanism seems to be exercised by *S. aureus*. Tan et al. identified *S. aureus* strains intra-cellularly and in the sub-mucosa of adult patients undergoing endoscopic sinus surgery for CRS. This mechanism could decrease the ability of penetration of many antimicrobials and thus increase bacterial resistance [27].

Other bacteria are certainly involved in forming pathogenic biofilms in patients with ARS or CRS, but further studies and more feasible methods are required to identify the real contribution of biofilms in the pathophysiology of CRS, especially in children. However, it is known that the main negative result in CRS patients with biofilm-endowed bacteria is a high degree of refractoriness to antibiotics and mucosal immunity as these bacteria are less susceptible to many environmental factors, antibiotics, and host immune mechanisms [3].

## 4. Lessons from the Adult Nasosinus Microbiome: Pediatric Implications

The term “microbiota” refers to the entire cohort of resident commensal, symbiotic, and pathogenic micro-organisms inhabiting a given niche (such as the sinus cavities) that are organized and act as a single community. Recent studies revealed the complex and highly variable nasopharyngeal microbiota in children, which undergo significant changes during disease, and after exposure to anti-microbial agents or vaccinations [28]. The nasal microbiota consists of various microbial communities, including many different genera of aerobic and anaerobic micro-organisms such as *Staphylococcus spp.*, *Corynebacterium spp.*, and *Propionibacterium spp.* [29] 

The disruption of indigenous microbiota (dysbiosis) may lead to pathogen overgrowth and greater susceptibility to infection, as has been observed in the gastrointestinal tract and lower airways. Resident microbes may also influence the behavior of pathogenic species in a “community as pathogen” model and further promote the development of rhinosinusitis [4].

In principle, exposure to greater biodiversity could increase competition in the nasal microbiota, and thus reduce colonization by opportunistic pathogens. A recent study by Shukla et al. demonstrated that the nasal microbiome among dairy farmers is more complex than their oral microbiota: it protects against infections and competes with *Staphylococcus aureus* colonization [30]. Another study of adults showed that an abundance of nasal coagulase-negative staphylococci can negatively interfere with and counteract *S. aureus* colonization [31]. 

The abundance and diversity of nasal microbiota are therefore prognostic of healthiness (Box 4). Biswas et al. [32] found that the diversity of bacterial communities was significantly less in samples taken from patients with CRS than in those taken from healthy subjects whose bacterial load was not associated with disease. The positive and negative effects of microbiota complexity in patients ARS or CRS have now been clearly documented [33,34,35], and this has recently led to new treatments such as “bacteriotherapy”, which exploits the ability of saprophytic bacteria to counteract colonization [36]. Furthermore, Roos et al. demonstrated that commensal α-hemolytic streptococci can be used to replace normal nasopharyngeal flora in children with recurrent infections [37].

Box 4 **Microbiota health depends on microbial diversity, abundance, and evenness.**

Sinonasal microbiota can be associated with statistically different bacterial communities in patients with ARS or CRS. Infants with more frequent symptomatic infections show less bacterial diversity.

It has recently been found that a combined *Streptococcus salivarius* 24SMBc and *Streptococcus oralis* 89a nasal spray can create a bio-barrier against pathogens, thus preventing their replication and restoring normal bacterial microbiota [38]. This promising approach could also be used to reduce potentially harmful bacteria and increase the abundance of beneficial bacteria in patients with rhinosinusitis. Abreu et al. found that Lactobacillus sakei can protect normal sinus microbiota in adult CRS patients and lead to a significant reduction in bacterial diversity. Interestingly, the same authors noted that the pathophysiology of CRS can also be negatively influenced by a relative abundance of Corynebacterium tuberculostearicum [39]. These findings were confirmed by a recent meta-analysis of studies of sinonasal microbiota in CRS patients, which not only showed that a greater abundance of members of the genus Corynebacterium can be associated with CRS, but also that the genera Burkholderia and Propionibacterium are potentially important gatekeepers that can maintain the balance of sinonasal microbiota [40]. 

Our knowledge of the microbiota to be found in CRS patients is still evolving but, although there have been many microbiome meta-genomic analyses of adults, only a few comparative studies of pediatric patients have been published. Pasha has suggested that there is a need for further longitudinal studies in order to evaluate the effects of anti-microbial agents, sinus surgery, and topical nasal treatments on microbial diversity and abundance in patients with CRS [41]. However, given the conflicting results of the studies that have already been published, it needs to be remembered that future studies of children should bear in mind the fact that interpersonal variations, biogeography, lifestyles (especially smoking), sampling methods, and local or systemic diseases can also greatly affect the results.

## 5. Conclusions

There are significant differences in the microbiological aspects of CRS and ARS. *Staphylococcus aureus*, *S. epidermidis*, and anaerobic Gram-negative bacteria predominate in CRS but, although persistent infection can promote the growth of anaerobic bacteria, their true prevalence has been misrepresented by studies that did not use appropriate recovery methods. 

The presence of a new acute pediatric rhinosinusitis where sinusitis developed in an anatomically sequestered cavity, an active/negative local microbiome and pathogen/s interaction (not necessarily endowed in a biofilm) can presumably lead to a rapid progression of the disease. Subsequently, without an appropriate antibiotic therapy and/or with continued lack of natural clearing of accumulated mucous, biofilmed or non-biofilmed pathogenic bacteria become entrapped, and after 8–12 weeks of incubation obtain sufficient bacterial overgrowth to result in a chronic rhinosinusitis. Most of all these processes involved in the acute “bacterially” infected sinus tract and in the development of a chronic rhinosinusitis are still not known.

Microbiome studies can lead the way to better clarify the specific pathways regulating the stability and resilience of a bacterial ecosystem, and to further develop strategies for ecological modulation especially in subjects with an altered microbiota. The relationship between microbiota and sinonasal diseases has been well characterized, but further studies are necessary in order to establish its implications for the bacterial network under pathological conditions.

## References

[1] Brook I. (1981). Bacteriologic features of chronic sinusitis in children. JAMA.

[2] Wald E.R., Byers C., Guerra N., Casselbrant M., Beste D. (1989). Subacute sinusitis in children. J. Pediatr..

[3] Hall-Stoodley L., Costerton J.W., Stoodley P. (2004). Bacterial biofilms: From the natural environment to infectious diseases. Nat. Rev. Microbiol..

[4] Jivianne T., Lee D., Frank N., Ramakrishnan V. (2016). Microbiome of the paranasal sinuses: Update and literature review. Am. J. Rhinol. Allergy.

[5] Muntz H.R., Lusk R.P. (1991). Bacteriology of the ethmoid bullae in children with chronic sinusitis. Arch. Otolaryngol. Head Neck Surg..

[6] Tinkleman D.G., Silk H.J. (1989). Clinical and bacteriologic features of chronic sinusitis in children. Am. J. Dis. Child..

[7] Brook I., Frazier E.H., Foote P.A. (1996). Microbiology of the transition from acute to chronic maxillary sinusitis. J. Med. Microbiol..

[8] Marom T., Alvarez-Fernandez P.E., Jennings K., Patel J.A., McCormick D.P., Chonmaitree T. (2014). Acute bacterial sinusitis complicating viral upper respiratory tract infection in young children. Ped. Infect. Dis. J..

[9] Brook I. (2005). The role of bacterial interference in otitis, sinusitis and tonsillitis. Otolaryngol. Head Neck Surg..

[10] Brook I. (2016). Microbiology of chronic rhinosinusitis. Eur. J. Clin. Microbiol. Infect. Dis..

[11] Russell J.H., Allison J.A., Brooks D. (2016). Fifty Years of Chronic Rhinosinusitis in Children: The Accepted, the Unknown, and Thoughts for the Future. Pediat. Allergy Immunol. Pulmonol..

[12] Ivanchenko O.A., Karpishchenko S.A., Kozlov R.S., Krechikova O.I., Otvagin I.V., Sopko O.N., Piskunov G.Z., Lopatin A.S. (2016). The microbiome of the maxillary sinus and middle nasal meatus in chronic rhinosinusitis. Rhinology.

[13] Mantovani K., Bisanha A.A., Demarco R.C., Tamashiro E., Martinez R., Anselmo-Lima W.T. (2010). Maxillary sinuses microbiology from patients with chronic rhinosinusitis. Braz. J. Otorhinolaryngol..

[14] Brook I. (2006). Bacteriology of chronic sinusitis and acute exacerbation of chronic sinusitis. Arch. Otolaryngol. Head Neck Surg..

[15] Brook I. (2017). The role of antibiotics in pediatric chronic rhinosinusitis. Laryngosc. Investig. Otolaryngol..

[16] Costerton J.W., Lewandowski Z., Caldwell D.E., Korber D.R., Lappin-Scott H.M. (1995). Microbial biofilms. Annu. Rev. Microbiol..

[17] Wood A.J., Fraser J.D., Swift S., Patterson-Emanuelson E.A., Amirapu S., Douglas R.G. (2012). Intramucosal bacterial microcolonies exist in chronic rhinosinusitis without inducing a local immune response. Am. J. Rhinol. Allergy.

[18] Pagella F., Colombo A., Gatti O., Giourgos G., Matti E. (2010). Rhinosinusitis and otitis media: The link with adenoids. Int. J. Immunopathol. Pharm..

[19] Coticchia J., Zuliani G., Coleman C., Carron M., Gurrola J., Haupert M. (2007). Biofilm Surface area in the pediatric nasopharynx. Arch. Otolaryngol. Head Neck Surg..

[20] Kania R.L., Lamers G., Vonk M., Dorpmans E., Struik J., Tran Ba Hui P., Hiemstra P., Bloemberg G., Grote J. (2008). Characterization of mucosal biofilms on human adenoid tissue. Laryngoscope.

[21] Mladinaa R., Skitarelić N. (2010). Biofilm*—*The other name for the regular mucosal blanket. Med. Hypotheses.

[22] Keir J., Pedelty L., Swift A.C. (2011). Biofilms in chronic rhinosinusitis: Systematic review and suggestions for future research. J. Laryngol. Otol..

[23] Torretta S., Drago L., Marchisio P., Mattina R., Clemente I.A., Pignataro L. (2011). Diagnostic accuracy of nasopharyngeal swab in biofilm detection in children with chronic adenoiditis: A preliminary study. Otolaryngol. Head Neck Surg..

[24] Starner T.D., Zhang N., Kim G., Apicella M.A., McCray P.B. (2006). Haemophilus influenza Forms Biofilms on Airway Epithelia: Implications in Cystic Fibrosis. Am. J. Resp. Crit. Care Med..

[25] Perloff J., Palmer J.N. (2005). Evidence of bacterial biofilms in a rabbit model of sinusitis. Am. J. Rhinol..

[26] Tapiainen T., Kujala T., Kaijalainen T., Ikäheimo I., Saukkoriipi A., Renko M., Salo J., Leinonen M., Uhari M. (2010). Biofilm formation by Streptococcus pneumoniae isolates from paediatric patients. APMIS.

[27] Tan N.C., Tran H.B., Foreman A., Jardeleza C., Vreugde S., Wormald P.J. (2012). Identifying intracellular Staphylococcus aureus in chronic rhinosinusitis: A direct comparison of techniques. Am. J. Rhinol. Allergy.

[28] Mika M., Mack I., Korten I., Qi W., Aebi S., Frey U., Latzin P., Hilty M. (2015). Dynamics of the nasal microbiota in infancy: A prospective cohort study. J. Allergy Clin. Immunol..

[29] Yan M., Pamp S.J., Fukuyama J., Hwang P.H., Cho D.Y., Holmes S., Relman D.A. (2013). Nasal microenvironments and interspecific interactions influence nasal microbiota complexity and *S. aureus* carriage. Cell Host Microbe.

[30] Shukla S.K., Ye Z., Sandberg S., Reyes I., Fritsche T.R., Keifer M. (2017). The nasal microbiota of dairy farmers is more complex than oral microbiota, reflects occupational exposure, and provides competition for staphylococci. PLoS ONE.

[31] Frank D.N., Feazel L.M., Bessesen M.T., Price C.S., Janoff E.N., Pace N.R. (2010). The Human Nasal Microbiota and Staphylococcus aureus Carriage. PLoS ONE.

[32] Biswas K., Hoggard M., Jain R., Taylor M.W., Douglas R.G. (2015). The nasal microbiota in health and disease: Variation within and between subjects. Front. Microbiol..

[33] Feazel L.M., Robertson C.E., Ramakrishnan V.R., Frank D.N. (2012). Microbiome complexity and Staphylococcus aureus in chronic rhinosinusitis. Laryngoscope.

[34] Aurora R., Chatterjee D., Hentzleman J., Prasad G., Sindwani R., Sanford T. (2013). Contrasting the microbiomes from healthy volunteers and patients with chronic rhinosinusitis. JAMA Otolaryngol. Head Neck Surg..

[35] Ramakrishnan V.R., Hauser L.J., Feazel L.M., Ir D., Robertson C.E., Frank D.N. (2015). Sinus microbiota varies among chronic rhinosinusitis phenotypes and predicts surgical outcome. J. Allergy Clin. Immunol..

[36] Huovinen P. (2001). Bacteriotherapy: The time has come. Bacterial interference is an increasingly attractive approach to prevention and therapy. BMJ.

[37] Roos K., Håkansson E.G., Holm S. (2001). Effect of recolonization with “interfering” alpha streptococci on recurrences of acute and secretory otitis media in children: Randomised placebo controlled trial. BMJ.

[38] Santagati M., Scillato M., Patanè F., Aiello C., Stefani S. (2012). Bacteriocin-producing oral streptococci and inhibition of respiratory pathogens. FEMS Immunol. Med. Microbiol..

[39] Abreu N.A., Nagalingam N.A., Song Y., Roediger F.C., Pletcher S.D., Goldberg A.N., Lynch S.V. (2012). Sinus microbiome diversity depletion and Corynebacterium tuberculostearicum enrichment mediates rhinosinusitis. Sci. Transl. Med..

[40] Wagner Mackenzie B., Waite D.W., Hoggard M., Douglas R.G., Taylor M.W., Biswas K. (2017). Bacterial community collapse: A meta-analysis of the sinonasal microbiota in chronic rhinosinusitis. Environ. Microbiol..

[41] Pasha M.A. (2018). State-of-the-Art Adult Chronic Rhinosinusitis Microbiome: Perspective for Future Studies in Pediatrics. Sinusitis.

